# Supplementary material for: Deciphering key processes controlling rainfall isotopic variability during extreme tropical cyclones
Source: Nat Commun. 2019 Sep 20;10:4321. doi: 10.1038/s41467-019-12062-3 (PMC6754435; doi:10.1038/s41467-019-12062-3)
Supplement: Supplementary file 2 — Description of Additional Supplementary Files [file 41467_2019_12062_MOESM2_ESM.pdf]

Description of Additional Supplementary Files

File name: Supplementary Data 1

Description: Hurricane Otto isotopic composition.

File name: Supplementary Data 2

Description: Isotopic composition of hurricanes Irma and Maria.

File name: Supplementary Data 3

Description: Isotopic composition in rainfall of Central Costa Rica.

File name: Supplementary Data 4

Description: Isotopic composition in rainfall of the Caribbean slope of Costa Rica.

File name: Supplementary Data 5

Description: Rainfall amount, stratiform fraction (Fst), and isotopic composition in the Caribbean slope of Costa Rica.
